# Cardiac Tamponade Masquerading as Lactic Acidosis

**DOI:** 10.1155/cric/6505753

**Published:** 2026-11-15

**Authors:** Ahad Firoz, Anne Tran, Tahir Mahmood, Amogh Bhat, Michael Schivo

**Affiliations:** ^1^ Department of Internal Medicine University of California Davis Health Sacramento California USA ucdavis.edu; ^2^ University of California Davis School of Medicine Sacramento California USA ucdavis.edu; ^3^ Division of Cardiovascular Medicine Department of Internal Medicine University of California Davis Health Sacramento California USA ucdavis.edu; ^4^ Division of Cardiovascular Medicine Department of Internal Medicine Sacramento Veterans Affairs Medical Center Mather California USA; ^5^ Division of Pulmonary and Critical Care Medicine Department of Internal Medicine University of California Davis Health Sacramento California USA ucdavis.edu; ^6^ Division of Pulmonary and Critical Care Medicine Department of Internal Medicine Sacramento Veterans Affairs Medical Center Mather California USA

## Abstract

An 85‐year‐old male presented with acute dyspnea and syncope, with pertinent initial vital signs including hypertension, normal heart rate, tachypnea, and hypoxemia requiring oxygen supplementation. The Medical Intensive Care Unit (MICU) team was consulted for treatment of acute hypoxic respiratory failure, severe hyperglycemia, and lactic acidosis; on MICU evaluation, bedside point of care ultrasonography (POCUS) revealed a large, circumferential pericardial effusion. Despite ongoing intravenous fluid resuscitation, the patient rapidly developed cardiac tamponade with pulsus paradoxus and hypotension. Given the severe hyperglycemia and likely resultant osmotic diuresis, his atypical presentation may have represented low‐pressure cardiac tamponade phenotype. He was emergently taken for pericardiocentesis and drain placement with immediate resolution of clinical tamponade; the etiology of his pericardial effusion was deemed idiopathic, although a hemorrhagic effusion from anticoagulation use cannot be ruled out. Ultimately, this case highlights POCUS as an essential clinical tool that should be utilized upon initial assessment of ICU level of care patients to avoid missing indolent but fatal conditions, such as developing or manifest cardiac tamponade. Once cardiac tamponade is identified, careful intravenous fluid resuscitation may be appropriate under the correct circumstances, although definitive treatment involves emergent pericardiocentesis.

## 1. Introduction

Point of care ultrasonography (POCUS) is a highly versatile tool that has gained widespread adoption across several medical specialties and is rapidly integrating into undergraduate medical education curriculums [1]. In addition to limiting patient radiation exposure, POCUS allows for rapid and real‐time bedside assessment and management of various clinical conditions. In particular, it can provide a thorough cardiovascular assessment and aid in identifying several high‐acuity pathologies, including ventricular dysfunction, volume overload, valvulopathies, and tamponade physiology. Our case report describes an atypical presentation of cardiac tamponade that was rapidly identified on admission by POCUS.

## 2. Case

An 85‐year‐old male with a pertinent past medical history of type 2 diabetes, hypertension, paroxysmal atrial fibrillation, chronic obstructive pulmonary disease (COPD), and chronic kidney disease presented with 1 day of dyspnea and dizziness, resulting in a syncopal episode. Additionally, he reported elevated blood sugar levels in the 400 s at home. Relevant home medications included dose‐reduced apixaban 2.5 mg twice daily, atorvastatin 40 mg once daily, empagliflozin 12.5 mg once daily, glimepiride 2 mg once daily, lisinopril 20 mg once daily, and metformin 500 mg twice daily. The indication for apixaban was for paroxysmal atrial fibrillation stroke prevention. The patient was compliant with his medications, and the last dosage of the apixaban was the evening prior to the day of presentation.

Initial vital signs were pertinent for hypertension (160s/60 s), hypoxemia with SpO_2_ of 84% requiring 6 L/min of supplemental oxygen via nasal cannula, tachypnea with respiratory rate 20–30 breaths per minute, and a normal heart rate of 91 beats per minute. The Emergency Department (ED) consulted the Medical Intensive Care Unit (MICU) team for admission for hypoxia, severe hyperglycemia, and lactic acidosis (LA), with concern for COPD exacerbation and diabetic ketoacidosis (DKA) as the primary underlying etiologies.

A comprehensive work‐up was done initially in the ED prior to formal evaluation by the MICU team. The complete metabolic panel was pertinent for severe hyperglycemia (533 mg/dL), acute on chronic kidney injury (creatinine of 3.1 mg/dL from baseline of 1.5 mg/dL), hyperkalemia (7.0 mmol/L), anion‐gap metabolic acidosis (bicarbonate: 8 mmol/L, anion‐gap: 22 mmol/L) with concomitant respiratory acidosis on venous blood gas (pH: 7.10, PaCO_2_: 26 mmHg), elevated liver enzymes in a hepatocellular pattern (AST: 423 U/L, ALT: 246 U/L), and elevated high sensitivity Troponin I (56 ng/L) and NT‐proBNP (2198 pg/mL). The Troponin I later returned within normal limits on a recheck approximately 2 hours later. An arterial blood gas that was collected while the patient was on non‐rebreather mask showed an elevated PaO_2_ of 227 mmHg. His lactate was significantly elevated at 12.4 mmol/L. Infectious work‐up was overall negative. Chest x‐ray was notable for emphysematous changes but was otherwise unremarkable with a normal sized cardiac silhouette, and without cardiomegaly, pulmonary edema, or consolidations. The electrocardiogram (Figure 1) demonstrated normal sinus rhythm with rate 91 beats per minute, first degree atrioventricular block, incomplete right bundle branch block, and new onset low voltage in all leads without any ischemic changes. Given the patient′s continued increased work of breathing, the ED placed the patient on high‐flow nasal cannula. Additionally, they initiated treatment with volume resuscitation and an insulin drip for presumed DKA, as well as empiric therapy for a COPD exacerbation with albuterol and ipratropium nebulizer treatment, 10 mg of intravenous dexamethasone, and 500 mg of azithromycin.

**Figure 1:**
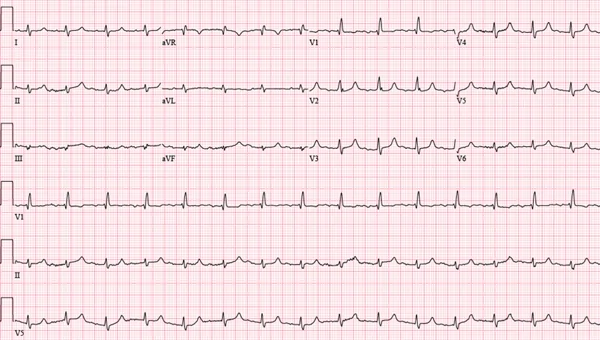
Electrocardiogram on initial presentation notable for new‐onset low‐amplitude voltage.

On initial encounter by the MICU team, the patient was noted to be tachypneic with dry mucous membranes. The pulmonary exam was notable for diffuse wheezing; cardiac examination was limited secondary to patient′s respiratory distress. Full assessment of jugular venous pressure and Kussmaul′s sign was precluded by the patient′s respiratory distress. Given his unexplained hypoxia, bedside POCUS was conducted which revealed a large, circumferential pericardial effusion (Figure 2; Videos S1 and S2) and plethoric inferior vena cava (IVC) with minimal respiratory variation (Video S3). Despite initial volume resuscitation for hypovolemia and concern for DKA, the patient′s blood pressure dropped precipitously during the course of evaluation, necessitating vasopressor support. Emergent arterial access was obtained for hemodynamic monitoring, which revealed significant pulsus paradoxus of ≥ 20 mmHg, and repeat bedside POCUS demonstrated concern for early systolic right atrial and late diastolic right ventricular collapse.

**Figure 2:**
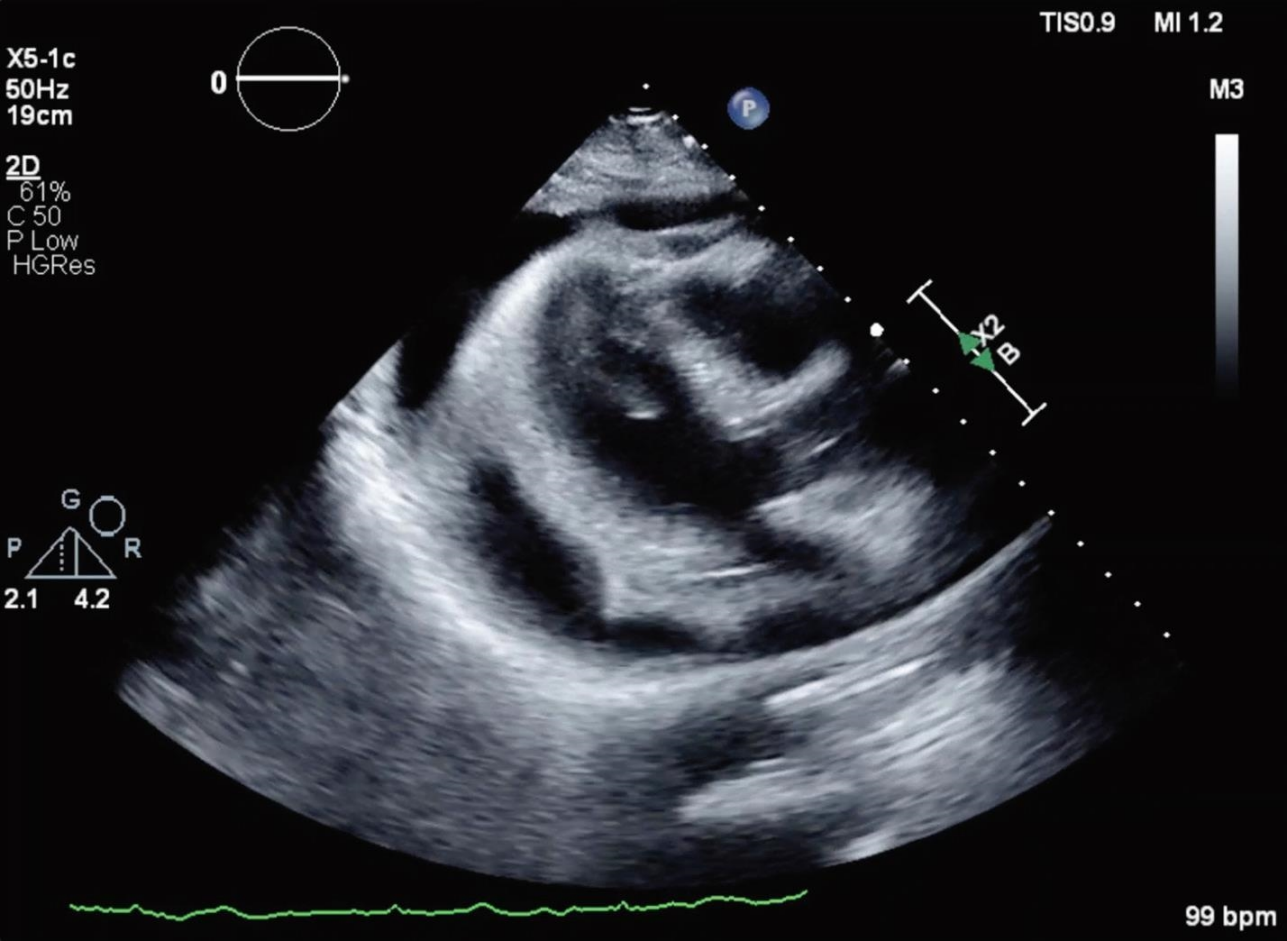
Transthoracic echocardiogram on hospital admission in the parasternal long axis view at end diastole illustrating a large circumferential pericardial effusion.

The patient was given 2000 units of prothrombin complex concentrate as a reversal agent for his anticoagulation and emergently taken to the cardiac catheterization laboratory for pericardiocentesis and pericardial drain placement. Approximately 800 cc of dark, sanguineous fluid was initially drained with immediate resolution of pulsus paradoxus and normalization of arterial pressures. Given the emergent nature of the procedure, simultaneous right heart catheterization was deferred. Subsequent transthoracic echocardiogram demonstrated trace circumferential pericardial effusion with normal right and left ventricular function without evidence of tamponade including normal IVC (Figure 3; Videos S4 and S5). Lactate levels decreased precipitously to 6.7 immediately after pericardiocentesis, and the LA fully resolved on a subsequent 3‐hour recheck. Urinalysis was negative for ketones, and DKA was definitively ruled out later in the hospitalization with beta‐hydroxybutyrate levels of 0.9 mg/dL. Several etiologies of pericardial effusion were assessed (Table 1), none of which revealed a specific etiology; thus the patient was diagnosed with idiopathic hemorrhagic pericardial effusion as the working diagnosis. Following the pericardiocentesis, his hemoglobin level resulted at 8.5 g/dL (approximate baseline level of 9–10 g/dL); as such, a blood transfusion was not immediately required. Although it is reasonable to surmise that his anticoagulation use may have predisposed him to the pericardial effusion, it is unclear how much of a role it played in its inception or acceleration.

**Figure 3:**
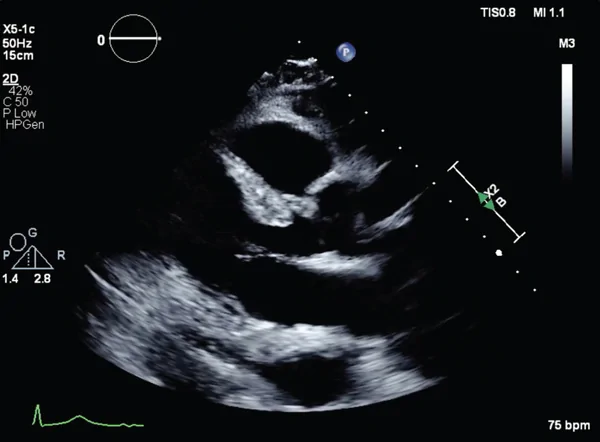
Transthoracic echocardiogram status‐post emergent pericardiocentesis in the parasternal long axis view at end diastole with pericardial drain in place.

**Table 1:** Work‐up for underlying etiology of pericardial effusion.

Work‐up	Results
Infectious etiologies	
Cultures^a^	No growth
Concentrate acid fast stain^a^	Negative
Respiratory viral panel	Negative
Cell count analysis^a^	

WBC	9240/mm^3^ (H)
Polys	83% (H)
Lymphs	6% (H)
Eosino	11% (H)
RBC	2380000/mm^3^ (H)
Hematocrit	27% (H)
Appearance	Turbid red
Pathologist review	No evidence of malignancy
Malignancy work‐up^b^	

Carcinoembryonic antigen	1.8 ng/mL
CA 19‐9	< 2 U/mL
Alpha fetoprotein	< 2 ng/mL
Autoimmune work‐up^b^	

Antinuclear antibody	< 1:80
Inflammatory work‐up^b^	

High sensitivity C‐reactive protein	4.7 mg/L

*Note:* Work‐up for underlying etiology of pericardial effusion was unremarkable, resulting in a working diagnosis of idiopathic hemorrhagic pericardial effusion.

^a^Pericardial fluid analysis.

^b^Serum analysis.

## 3. Outcome and Follow‐Up

The patient initially had minimal output from the pericardial drain. On postoperative day 3, his hospitalization course was complicated by catheter‐associated thrombus requiring intraluminal thrombolytics. Ultimately, the pericardial drain was removed after 5 days without need for pericardial window. The patient was discharged with a 3 month prescription of colchicine for presumed pericarditis. The patient subsequently had outpatient follow up with his primary care provider and cardiologist and reported feeling back to his baseline without recurrence of symptoms. Repeat transthoracic echocardiogram 1 month after hospital discharge demonstrated trace pericardial effusion without significant pathologic changes.

## 4. Discussion

Pericardial effusions are defined as an accumulation of > 50 mL of fluid in the pericardial space [2]. The early classification system for pericardial effusions was based on absolute fluid volume (small 50–100 mL, moderate 100–500 mL, large > 500 mL). More recently, the American College of Cardiology guidelines define pericardial effusion size based on the echocardiographic measurement of the greatest diameter perpendicular to the epicardium and parietal pericardium at end‐diastole. Effusions that are < 1.0 cm but visualized throughout the cardiac cycle are considered small; 1.0–1.9 cm are moderate; 2.0–2.5 cm are large; and > 2.5 cm are very large [3]. Common causes of pericardial effusions vary by geographic region. Idiopathic pericarditis, presumed to be postviral, is the leading etiology in North America and Western Europe and accounts for approximately half of cases, whereas tuberculosis may constitute 65%–70% of cases in endemic regions [3–5]. Other causes include malignancies, autoimmune diseases, and radiation therapy. Of the different etiologies, malignant effusions have been observed to be the predominant cause of tamponade, with lung cancer being the most common [6]. Approximately 3% of patients with pericardial effusions will have tamponade physiology on echocardiography [2].

Cardiac tamponade is a hemodynamic, clinical diagnosis classically described as Beck′s triad of hypotension, jugular vein distention (JVD), and muffled heart sounds. In clinical practice, tamponade patients rarely present with the complete triad [7]. Rather, the more common findings include dyspnea, cardiomegaly, sinus tachycardia, pulsus paradoxus, and elevated jugular venous pressure [8]. Clinical symptoms can be correlated with echocardiographic findings to help establish the diagnosis. Given the obstructive shock physiology that ensues with cardiac tamponade, prompt recognition is vital to identify and manage this critical condition. POCUS, which is now readily available at many institutions, may allow clinicians to rapidly identify cardiac tamponade without having to wait for a formal echocardiogram. A dilated inferior vena cava (> 2.1 cm) with minimal or no change during respiration is a highly sensitive marker for cardiac tamponade, whereas early diastolic right ventricular collapse is the most specific finding [3]. For patients with unfavorable body habitus, POCUS can also be utilized as a means to identify JVD using the vascular probe. As a surrogate for pulsus paradoxus, doppler flow velocity will typically display a respiratory variation of > 30% in mitral peak E inflow or > 60% in peak tricuspid E inflow [2]. Within the broad category of cardiac tamponade, there are several rare phenotypes that have been observed.

Low‐pressure cardiac tamponade is an uncommon subtype that is more difficult to clinically recognize. This occult condition is thought to occur in patients with pericardial effusions who are hypovolemic, often in the setting of diuresis, blood loss, or hemodialysis, causing relatively low pericardial pressures to exceed intracardiac filling pressures. Most patients with low‐pressure cardiac tamponade do not present with classic symptoms; however, they do demonstrate a significant increase in cardiac output after pericardiocentesis [9]. Although no strict diagnostic criteria for low‐pressure tamponade have been readily established, Sagristà‐Sauleda et al. developed criteria measurements that are commonly utilized in the literature for this rare phenotype; namely, intrapericardial pressures < 7 mmHg prior to pericardiocentesis, followed by right atrial pressures < 4 mmHg after pericardial fluid drainage [9]. Using these guidelines in their retrospective analysis, the authors discovered that approximately 20% of their cohort meeting criteria for cardiac tamponade had a low‐pressure physiology [9].

Interestingly, despite being in a hypovolemic state, several case reports have described limited immediate benefit in volume resuscitation for low‐pressure tamponade [10–14]. A similar phenomenon was observed in an investigation that analyzed the effect of volume expansion in cardiac tamponade; in this study, the authors discovered that right atrial pressures during cardiac tamponade were not predictive of a significant increase in cardiac index with volume resuscitation [15]. Interestingly, in the same study, approximately 31% of patients had a decrease in their cardiac index after they received a 500 cc fluid bolus [15]. Although the exact mechanism remains unclear and poorly understood, other cases have found volume resuscitation to unmask classical tamponade [10, 11, 16]. Ultimately, pericardiocentesis is the definitive treatment for tamponade, resulting in immediate hemodynamic stabilization.

In the case of our patient, although the true chronicity of the effusion is unknown, chest computed tomography without contrast that was obtained as part of a pulmonary workup several months prior to presentation did not demonstrate evidence of pericardial effusion, suggesting a subacute to chronic process. Given his severe hyperglycemia and presumed prerenal acute kidney injury, there may have been a component of osmotic diuresis, which could have precipitated his effusion into a low‐pressure tamponade physiology; notably, this combination of decreased preload with reduced cardiac output may have induced his syncopal episode at home. Despite having an elevated blood pressure without signs of classic tamponade on presentation, he had a severe LA without ketosis, suggesting hypoperfusion presumably from impaired cardiac output from indolent low‐pressure tamponade. However, after volume resuscitation, he rapidly developed markers of classic tamponade, including plethoric IVC, pulsus paradoxus, and eventual hypotension. These emergent findings, including the hypoxia, severe LA, and cardiac tamponade, all resolved following pericardiocentesis. Nonetheless, our investigation is limited by a lack of intrapericardial and intracardiac pressures at the time of emergent pericardiocentesis due to unstable hemodynamics. However, this limitation would not have changed management and may not have shown evidence of low‐pressure tamponade given presumably adequate intravascular volume resuscitation by the time of pericardiocentesis. Additionally, the initial LA could be confounded by the concurrent use of metformin in the setting of an acute kidney injury, although it would be unusual for such a dramatic drop in lactate levels following an intervention for presumed type A LA. Regardless, patients with multiple predisposing factors for both a type A and B LA should be treated empirically for the underlying etiology responsible for a type A LA, in this case being cardiac tamponade, which resulted in resolution of the LA.

## 5. Conclusion

Our case report describes an insidious and atypical presentation of cardiac tamponade that would not have been identified without the routine use of POCUS. In these cases, particularly when there is clinical evidence of hypovolemia, low‐pressure cardiac tamponade should be considered. Once cardiac tamponade is identified, careful intravenous fluid resuscitation may be appropriate under the correct circumstances, although definitive treatment involves emergent pericardiocentesis. In short, this case highlights early POCUS as an essential clinical tool that should be utilized upon initial assessment of ICU level of care patients, as it can rapidly identify life‐threatening diagnoses, such as cardiac tamponade, even in atypical presentations.

## Funding

No funding was received for the preparation of this manuscript.

## Conflicts of Interest

The authors declare no conflicts of interest.

## Supporting Information

Additional supporting information can be found online in the Supporting Information section.

## Supporting information


**Supporting Information 1** Video S1: Transthoracic echocardiogram on hospital admission in the parasternal long axis view illustrating a large, circumferential pericardial effusion.

**Supporting Information 2** Video S2: Transthoracic echocardiogram on hospital admission in the subxiphoid view illustrating a large, circumferential pericardial effusion.

**Supporting Information 3** Video S3: Transthoracic echocardiogram on hospital admission demonstrating a plethoric inferior vena cava with minimal to no respiratory variation.

**Supporting Information 4** Video S4: Transthoracic echocardiogram status‐post emergent pericardiocentesis in the parasternal long axis view demonstrating trace circumferential pericardial effusion with intact ventricular function.

**Supporting Information 5** Video S5: Transthoracic echocardiogram status‐post emergent pericardiocentesis in the subxiphoid view illustrating trace circumferential pericardial effusion with intact ventricular function.

## Data Availability

Data sharing is not applicable to this article as no datasets were generated or analyzed during the current study.
